# Evans Blue Dye: A Revisit of Its Applications in Biomedicine

**DOI:** 10.1155/2018/7628037

**Published:** 2018-04-22

**Authors:** Linpeng Yao, Xing Xue, Peipei Yu, Yicheng Ni, Feng Chen

**Affiliations:** ^1^Department of Radiology, The First Affiliated Hospital, Zhejiang University School of Medicine, 79 Qingchun Road, Hangzhou, Zhejiang 310003, China; ^2^Department of Radiology, Sanmen County People's Hospital, Sanmen, Zhejiang 317100, China; ^3^Radiology Section, University Hospitals, University of Leuven, 3000 Leuven, Belgium

## Abstract

Evans blue (EB) dye has owned a long history as a biological dye and diagnostic agent since its first staining application by Herbert McLean Evans in 1914. Due to its high water solubility and slow excretion, as well as its tight binding to serum albumin, EB has been widely used in biomedicine, including its use in estimating blood volume and vascular permeability, detecting lymph nodes, and localizing the tumor lesions. Recently, a series of EB derivatives have been labeled with PET isotopes and can be used as theranostics with a broad potential due to their improved half-life in the blood and reduced release. Some of EB derivatives have even been used in translational applications in clinics. In addition, a novel necrosis-avid feature of EB has recently been reported in some preclinical animal studies. Given all these interesting and important advances in EB study, a comprehensive revisiting of EB has been made in its biomedical applications in the review.

## 1. Introduction

Dating back to the early 20th century, lots of blue dyes were synthesized like Methylene blue, Patent blue, Trypan blue, and so forth, and a comparison of main blue dyes was given in Table 1 [1–4]. Among those, Evans blue (EB) dye is just one that has a long history as a biological dye and clinical diagnostic agent [5]. As a synthetic bis-azo dye, EB dye is also named T-1824 and Direct Blue 53. Differently, with the first staining application initiated by Herbert McLean Evans in 1914, this dye became famous when it was sold by the Eastman Kodak Company under the name of “Evans blue” in recognition of Mr. Evan's contributions to the development and use of dyes since 1936 [5, 6]. From that moment, the name “Evans blue” was widely accepted.

EB dye, with a molecular weight of 961 Da and high water solubility (280 g/l), strongly binds to serum albumin* in vivo* and* in vitro* and, thereby, becomes a high molecular weight protein tracer (69 kDa) [7–10]. When injected intravenously or intraperitoneally, albumin-bound EB remains stable in the blood and distributes throughout the entire body. Once injected in excess, EB dye can stain an entire animal including eyes, ears, nose, and paws with an intense blue color [11, 12].

In addition, Steinwall and Klatzo in 1966 reported that EB dye can emit a bright red fluorescence when activated with green light [7]. Thus, the dye provides a simple and convenient way of identification both macroscopically and microscopically using fluorescence for both morphological investigation and quantification. The fluorescent feature of EB dye has been used in various aspects including the characterization of the lymph nodes that drain the liver [13], confirmation of a satisfactory injection into the heart chambers of a zebrafish embryo [14], and observation of the extravasation of the dye from blood vessels into tissues [15–17].

Due to its high water solubility and slow excretion, as well as its firm binding to serum albumin, EB is also widely used in biomedicine including the estimation of blood volume, the assessment of vascular permeability to macromolecules, the detection of lymph nodes, and the location of the tumor lesions [12, 18–24].

More recently, a series of EB derivatives have been labeled with PET isotopes and can be used as theranostics with broad potential applications [25, 26]. EB also has a necrosis-avid feature as EB-bound albumin preferentially enters damaged cells, but not healthy cells, and persists within the damaged cells [14]. Thus, EB can be used as an agent for tissue viability assessment. This article reviews the features of EB and its potential applications in biomedicine.

## 2. Applications Derived from Albumin-Binding Characteristic of Evans Blue Dye

The strong binding capacity of EB dye to serum albumin has been demonstrated and this property has become widely accepted [7, 8, 10]. Tsopelas and Sutton investigated a series of 20 sulfonic acid dyes in 2002 and elaborated that protein binding ability was correlated with dye molecular structure. He demonstrated that the EB dye with tetrasulfonic acid group yielded 70% protein binding affinities (Table 1) [27]. Moreover, sulfonation reaction between sulfonic acid group of dye and amino group on protein surface was responsible for the dye-protein binding mechanism.

In various species, the binding capacity of albumin appears to be strongest in humans and dogs as 8–14 moles of Evans blue [28]. When injected intravenously, EB becomes fully albumin-bound and only a small proportion of free EB (0.11%–0.31%) can be found in the blood. The fact that EB dye is confined to the blood makes it unique among other dyes, for example, sodium fluorescein, which evenly distributes between the blood and organs such as the liver [10].

EB is eliminated from the circulating blood by the liver but not kidneys over time, which avoids the coloration of urine [27]. Wolman et al. [8] briefly reported the gradual disappearance of EB from the plasma and found that the EB in the plasma dropped rapidly over 24 h and remained very low in the blood after 3 days and was hardly detectable in their experiments. Importantly, as its unique characteristic, EB-protein complex with a high molecular weight may prevent its passage into living cells or across the blood-brain barrier. This leads to a variety of clinical applications that will be elaborated later in this article [29–31].

### 2.1. Use of Evans Blue Dye for Estimation of Blood Volume

The evaluation of changes in total blood volume is an interesting topic. The use of EB dye for this purpose was introduced by Keith et al. in 1915 [28, 34]. Earlier researchers in the field used red dyes from this class such as vital red, new vital red, and Congo Red. Until 1920, EB dye was considered superior to all the red dyes based on the investigations of Dawson et al., whose working principle of EB dye is now briefly described [35].

When a known quantity of dye (*N*) is injected into the blood stream, after an interval required for complete mixing, the quantity of dye (*n*) in 1 ml of withdrawn blood sample is analyzed. According to the relationship *V* = *N*/*n*, the plasma volume (*V*) can be calculated [36]. Then the total blood volume can be correctly calculated from the plasma volume according to the value of the hematocrit. Clearly, the only measurement that can be influenced by vital processes is that of *n*.

The dye dilution method using EB dye to measure plasma volume gained wide acceptance in the middle of the 20th century [37]. A wide variety of improvements and extraction methods were studied by researchers from various countries [34, 38–42]. To date, quantitation of EB dye can be optimized in limited tissue samples as much as 30 *μ*l [43]. However, since radioisotopic method to measure plasma volume was first introduced by Gibson and his coworkers in 1946, it has gradually replaced the EB dye dilution method [36, 44]. Compared with the dye method (which occasionally overestimates the plasma volume), ^131^I-labeled protein has been confirmed as an accurate measurement of plasma volume in both dogs and human subjects [45]. However, the method has its shortcomings due to the prolonged stay of radiolabeled material in the body; for example, repeated studies are not suggested on the same patient. Therefore, other methods such as trivalent chromium [^51^Cr(III)], hemoglobin-based oxygen carriers (HBOC), and carbon monoxide have been investigated as potential methods to estimate plasma volume [46, 47].

The carbon monoxide method appeared to be promising, especially in critically ill patients [46]. Yet et al. [47] developed a novel approach using HBOC in rabbits in 2001, which is inexpensive and not time-consuming, while Baby et al. [45] used ^51^Cr(III) in rabbits in 2014 and found that it was easy and quick and could be performed repeatedly in the clinical setting as ^51^Cr(III) is not bound to albumin or red blood cells (RBCs). To some extent, the Evans blue dye method to measure plasma and blood volumes is limited in animals, but, in humans, ^51^Cr(III) and HBOC are more promising for future clinical applications [48].

### 2.2. Use of Evans Blue Dye for Determination of Cardiac Output

The measurement of cardiac output by the dye injection method was established by Stewart [49] and later modified by Hamilton et al. [50]. Evans blue dye, as a vital stain, had been widely used in this aspect and the validity of the EB dye injection method has been shown to correlate well with the Fick procedure under different experimental conditions in dogs and humans [50, 51]. However, because of the slow excretion and undesirable discoloration, the EB dye was limited when repeated injections in patients were required. By contrast, Lacy et al. [20] demonstrated indigo carmine as an alternative to EB due to its firm binding to albumin and rapid removal by the liver and kidneys in 1955. In addition, Davis et al. [1] investigated a series of blue dyes in 1958 and found three rapid bloodstream clearance rates of dyes suitable for frequent serial estimation of the cardiac output. These included Patent blue A, Brilliant blue, and Patent blue V. However, owing to the invasiveness of the Fick technique and the dye injection method, they have been replaced by other more modern and simplified techniques including thermodilution techniques, lithium dilution, bioreactance, and Doppler technique or echocardiography [52, 53]. A few detailed reviews on this topic can be referred [52–54]. Therefore, EB and other blue dyes are no longer suitable for the determination of cardiac output except when used as a reference.

### 2.3. Use of Blood Pool Effect of Evans Blue Dye

EB dye is a permeable dye that can be easily taken up by degenerated or damaged cells but not by cells with an intact membrane [14]. However, interestingly, EB dye and triphenyl tetrazolium chloride (TTC) ex vivo double-staining are usually used together to assess the infarct size in acute myocardial infarction in both canine and murine models, where EB is delivered to the coronary arteries and TTC is applied to stain the surface of cardiac sections [47, 55–59]. When perfused into the aorta and coronary arteries in a retrograde manner, EB dye can uniformly distribute to everywhere in the vascular bed of normal myocardium, while infarcted myocardium or areas at risk cannot be stained due to the occlusion of the affected coronary arterial branch. The rationale behind TTC staining is that infarcted myocardium has lost dehydrogenase so that the colorless dead tissue on TTC cannot be converted to a red formazan [60]. Thus, the nonischemic area is stained blue, while the viable myocardium in the area at risk is stained red, and the infarcted myocardium appears to be pale [47].

EB dye can also be used alone to delineate which region of myocardium is perfused or hypoperfused based on accessibility to the dye [58]. But it seems to be paradoxical that the EB-perfused area, which is not at risk, is totally different from EB-stained cardiomyocytes that experience injury [61]. Indeed, due to the blood pool effect of EB, when perfused ex vivo, the EB-perfused area can stain rapidly following the branches of coronary artery. In contrast, the EB-staining of necrotic cardiomyocytes is due to the dye's affinity for dead cells, which will be addressed. Therefore, it is rational to say that EB dye could have a dual function, that is, assessment of the myocardial area at risk and assessment of infarcted myocardium.

### 2.4. Use of Evans Blue Dye for Assessment of Vascular Permeability

Integration of selective permeable barriers is vital for proper organ functioning and the maintenance of homeostasis. Such barriers include the endothelial cell barrier of blood vessels, the blood-brain barrier (BBB), the blood-retinal barrier, the blood-spinal cord barrier, and the blood-placental barrier [62]. The majority of selective permeable barriers are constituted primarily of tight junctions between endothelial cells to maintain barrier integrity. Under physiologic conditions, selective permeable barriers permit a selective transport of micromolecular substances but are impermeable to macromolecules such as albumin [63]. Thus, the EB dye, which strongly binds to albumin, remains restricted within the blood circulation and cannot cross the barrier. When various diseases result in a disruption of the barrier and increased vascular permeability, EB-bounded albumin may extravasate from the circulation into neighboring tissues. Leak of dye across the BBB or blood vessel signifies a disintegration of the barrier, and the accumulation of EB dye can be quantified after extraction from the stained tissue. The advantage of using EB dye as a tracer is its ability to be identified macroscopically, which gives a gross indication of its distribution. Furthermore, the extravasated EB dye within tissue can be observed by red fluorescence in tissue sections by fluorescence microscopy and quantified by colorimetry, spectrophotometry, and spectrophotofluorometry [62, 64, 65]. Thus, EB dye is extensively used to assess vascular permeability and BBB permeability described in various models, such as the classical Miles assay [24, 66, 67], the endothelial damage model caused by trauma [68], the stroke model [69], the cerebral ischemic model [64], and the breast cancer brain metastasis model [30].

In addition, Soubeyrand et al. [70] and Pettersson et al. [71] used EB dye to measure the permeability of the blood-spinal cord barrier and Xu et al. [72] detected the blood-retinal barrier breakdown by quantification using EB. It is worth noting that the stain administration route by intraperitoneal and intravenous injection had no difference on the amount of EB dye stain accumulated but a strong time-dependent tendency towards increase in stain accumulation [73]. As a rapid and inexpensive method, EB dye is still the most commonly used marker of brain barrier integrity and vascular permeability; however, its associated problems of tissue discoloration and potential toxicity need to be considered.

### 2.5. Use of Evans Blue Dye for Detection of Lymph Nodes

Visualization of the lymphatic system plays a significant role in assessing various malignancies and immune responses to foreign antigens in humans and in animal models [18]. The use of EB dye has become a major method for mapping lymphatic drainage. Due to the conjugation of the dye to plasma proteins by sulfonation reaction, the complex is sufficiently large to be trapped inside the lymphatic lumen and transported along with lymphatic flow [27]. Upon subcutaneous administration, the dye is quickly taken up by the lymphatic vessels to the draining nodes. Once the lymphatic mapping is shown by the blue color of EB dye, subsequent sentinel node biopsy becomes possible, which is critical for studies of solid tumor metastasis and of regional immune responses following immunization [18, 74].

Due to this mechanism of action, Bobin et al. [75] were able to evaluate the feasibility of sentinel lymph node identification in 100 breast cancer patients and confirmed the sensitivity of this technique (95%) in detecting nodal metastases. Harrell et al. [18] accurately and rapidly identified lymph nodes and lymphatic drainage in the mouse by injecting the dye into the mouse footpad or tail. Zheng et al. [13] successfully characterized the liver-draining lymph nodes by intrahepatic injection. Similarly, Tervala et al. [76] detected axillary lymph nodes and grafted lymph nodes by injecting EB dye solution intradermally. In addition, they analyzed the lymphatic vessel function by the way of quantifying the leakage of EB dye into the blood.

Except the EB dye, several other dyes are useful for identification of sentinel lymph nodes and include Chicago sky blue, Patent blue, and Trypan blue [27, 77]. It was previously reported that when scintigraphy and EB dye are used in tandem, the false-negative rate for sentinel node localization is decreased compared with using either agent alone [77]. On the whole, EB dye is a promising method for the assessment of malignancy and lymphedema in humans and subsequent sentinel node biopsy, as well as the evaluation of lymphatic vessel function.

### 2.6. Use of Evans Blue Dye for the Diagnosis and Identification of Tumors

Most tumors are known to exhibit highly enhanced vascular permeability, which is considered to facilitate tumor growth and perhaps metastasis [12, 15]. Visual demarcation of tumor margins during operation will provide greater accuracy in tumor resection. The use of a visible small molecular dye to address this challenge was suggested by Ozawa et al. [78]. EB dye is perfectly in accord with this demand, because EB dye may bind strongly to albumin, extravasates, and remains for a prolonged time in the extravascular space due to the enhanced permeability and retention (EPR) effect of tumors [79].

Lots of articles using EB dye to demarcate tumor margins were published [12, 30, 80, 81]. Prabhu et al. [80] calculated the uptake of the dye within an intracranial tumor to determine the volume of tumor and demonstrated that the method showed good correlation with the volume estimation from histological sections and gadolinium-enhanced magnetic resonance imaging. Likewise, a study by Elsen et al. investigated the biodistribution of Evans blue in a normal rat bladder and a bladder carcinoma and they found a significant difference of EB absorption between malignant tissues and normal bladder tissues after intravesical instillations [81]. Thus, the method of EB instillations combined with white-light cystoscopy could be a useful tool for diagnosing bladder cancer in clinical settings in the future.

Interestingly, by developing a liposomally nanoencapsulated Evans blue dye (nano-EB), Roller [12] demonstrated that clearer tumor margins were demarcated with nano-EB in an invasive tumor model compared with unencapsulated EB. This indicated that nano-EB could be deposited specifically to tumor tissue, which is normally considered as the tumor-specific feature of the EB dye [12, 82].

As we know, liposomal nanocarriers as drug carriers have been extensively investigated in nanomedicine; the special structure of liposomes can facilitate itself, evading the reticuloendothelial system and prolonging the circulation time in the bloodstream [83, 84]. Much effort has been made to design and optimize liposomal nanocarriers. For instance, incorporation of targeting moieties to tumors onto the liposomal may help to reduce the accumulation in nontarget organs [84]. Therefore, nano-EB may be used to provide accurate visual cues for the surgeon to intraoperatively delineate the tumor margins via two mechanisms: either via tumor EPR effect or via tumor-receptor targeting strategy [12].

In general, EB dye, especially liposomal nanocarriers-encapsulated Evans blue, could be a useful agent to localize and demarcate the solid tumors more precisely due to its selective accumulation in the tumor site. This so-called tumor-specific feature may actually be attributed to the EPR effect that may be used to explain the mechanism of EB applications in many clinical settings.

## 3. Use of Evans Blue Dye as a Necrosis-Avid Agent

As we know, life and death are two forms of cellular existence. Cell death is an essential form in cell development. Cell death includes at least two independent modes, that is, apoptosis and necrosis. EB dye was first reported by Gaff and Okong-O'Ogola as a vital stain for plant cells in 1971 [85]. In their study, EB dye leaked through damaged membranes and stained the dead cells but was excluded by viable cells.

EB dye has been further investigated and used extensively for specifying cell death in microscopic studies [86]. Although EB dye has been shown to be reliable for the assessment of viability in plant cells, it remains unclear whether EB dye can be also used in mammalian cells. In 1995, Matsuda et al. [87] attempted a new method using EB dye to identify degenerated muscle fibers in the mdx mouse and showed that EB-stained muscle fibers were either hypercontracted or degrading, which was consistent with the terminal-deoxynucleotidyl transferase mediated nick end labeling- (TUNEL-) positive myonuclei as supportive evidence for apoptosis. In contrast, a study by Straub et al. in 1997 identified that muscular fibers showing EB dye staining under fluorescence microscopy were necrotic fibers [88]. Similarly, Miller et al. [61] used EB dye to stain cardiomyocytes induced by myocardial contrast echocardiography in rats in 2007 and showed that the EB-stained cardiomyocytes had no TUNEL-positive nuclei after 24 h, which is evidence for necrosis instead of apoptosis. Klyen et al. also confirmed that the area of EB dye accumulation was consistent with the necrotic myofibers shown on hematoxylin and eosin staining and optical coherence tomography images [89].

**Figure 1:**
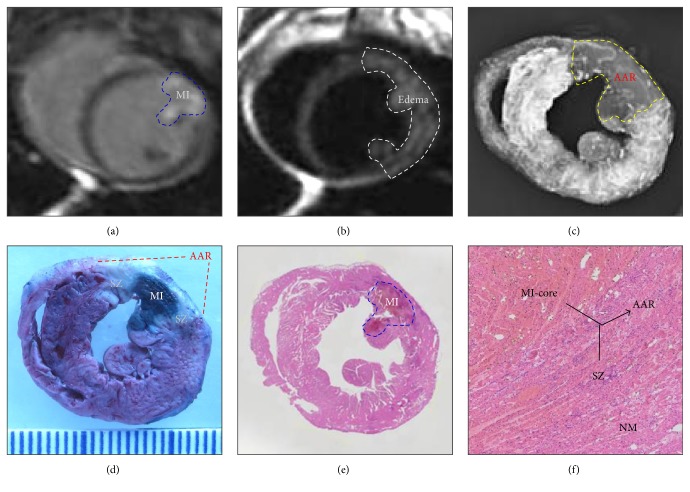
Evaluation of myocardial infarction core (MI-core), area at risk (AAR), and salvageable zone (SZ) in a rabbit with reperfused MI by* in vivo* and ex vivo imaging techniques and dynamic imaging quantification [32]. (a) Delayed, enhanced cardiac magnetic resonance imaging displays the MI-core as a transmural hyperenhanced area involving anterior papillary muscle; (b) T2-weighted imaging shows an extensive hyperintense region in the anterolateral wall; (c) digital radiograph of the red iodized oil-stained heart section shows a filling defect with few collateral vessels in the anterolateral wall in contrast to the rest of opaque left ventricle; (d) photograph of the heart section stained by multifunctional staining depicts the MI-core as an Evans blue dye-stained blue lesion simulating what is seen in (a) and shows the normal ventricular wall in red leaving the AAR (including the blue MI-core) unstained, which perfectly matches with the AAR in (c) and whitish zones which are suggestive of the SZ; (e) photomacroscopy of HE-stained heart slice views the MI-core as a hemorrhagic infarct similar in size to the blue lesion in (f); (f) photomicroscopy (×100) of HE-stained heart slice confirms the presence of the AAR (necrotic MI-core plus the viable but inflammatory SZ) and remote normal myocardium (NM) (reprinted and modified with permission from Feng Y, Chen F, Ma Z, Dekeyzer F, Yu J, Xie Y, Cona MM, Oyen R, Ni Y.* Theranostics*. 2013, 4: 24–35).

**Figure 2:**
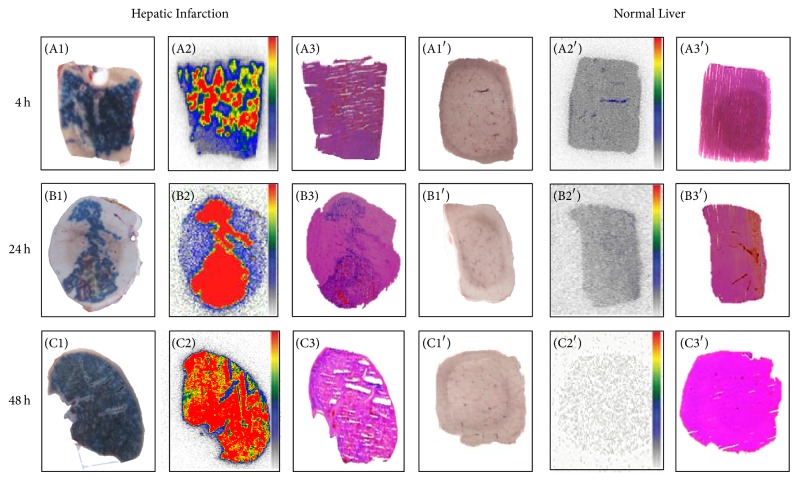
Postmortem analysis of necrotic and viable liver from rats with reperfused partial liver infarction that received iodine-123-labeled monoiodohypericin followed by the necrosis-avid dye, Evans blue [33]. At 4, 24, and 48 hours (h) after radioactivity injection, liver necrosis is outlined by the Evans blue as a blue region (A1, B1, and C1), with viable liver without staining (A1′, B1′, and C1′). Autoradiograms of 50 *μ*m thick sections show higher tracer accumulation in the hepatic infarction (A2, B2, and C2) than in viable liver (A2′, B2′, and C2′). The color code bar represents the coding scheme for the radioactivity. On histologic sections, the presence of scattered liver necrosis (A3, B3, and C3) and the location of the normal liver (A3′, B3′, and C3′) are confirmed (reprinted and modified with permission from Miranda Cona M, Koole M, Feng Y, Liu Y, Verbruggen A, Oyen R, Ni Y.* International Journal of Oncology *2014, 44: 819–829).

Many researchers used EB dye to discriminate among injured cells in various animal models, such as the zebrafish models of neuromuscular disease [14], mouse models of muscular dystrophy [90] and experimental injury and repair [91], rat models of stroke and cardiomyocyte injury [92], myocardial infarction core in a rabbit model [32] (Figure 1), and reperfused partial liver infarction model in rats [33] (Figure 2).

Furthermore, some studies have shown that EB's derivatives, such as EB-DTPA-Gd, have a specific binding affinity to a vascular lesion with endothelial damage, which is independent of serum proteins or the blood stream. EB-DTPA-Gd was also found to have a high affinity for atherosclerotic plaques* in vivo* in ApoE–/– mice [93]. These findings implied the nature of necrosis-avidity for EB dye. Although the EB dye was considered as a necrosis-avid agent, the specific mechanism is still not clear [94].

## 4. Radiolabeled Evans Blue Derivatives as Theranostics

In general, radiolabeling EB, such as in ^99m^Tc-EB, scarcely alters the pharmacodynamic properties of EB itself. ^99m^Tc-EB demonstrated that its affinity for plasma protein was not significantly different from that of unlabeled EB and both agents had similar dynamics regarding the sentinel lymph node [27, 74]. With the advantages of both radioactive and color signals in a single dose, it appears to be superior to the dual-injection technique of radiocolloid/blue dye in mapping the lymphatic system and discriminating sentinel lymph nodes [23, 95]. Therefore, ^99m^Tc-EB could be a promising agent in the clinical setting to guide sentinel node biopsy in various solid tumors in the near future.

Taking full advantage of the albumin-binding feature of EB, Liu et al. developed a number of radiolabeled EB derivatives [25, 26, 96–98]. These EB derivatives may be conjugated onto a therapeutic small molecule, peptide, or an oligonucleotide aptamer. Thus, they may be used as theranostics with a broad potential due to their improved half-life in the blood and reduced release. For instance, a version of truncated EB conjugated to 1,4,7-triazacyclononane-N,N′N′′-triacetic acid (NOTA) has been recently synthesized, named NEB. The NEB can be labelled with different PET isotopes including ^18^F-AlF, ^64^Cu, and ^68^Ga [96, 97]. These radiolabeled NEB derivatives were initially used as blood pool agents to evaluate myocardial infarction and vessel leakage in inflammation and tumors [96]. After local injection, ^18^F-AlF-NEB can be used to visualize and detect the sentinel lymph nodes with a high signal-to-noise ratio. More interestingly, as a blood pool imaging agent and PET tracer, ^68^Ga-NEB has already had translational applications in clinics. A preliminary study showed the potential of ^68^Ga-NEB in differentiating hepatic hemangioma from other focal hepatic lesions [97]. ^68^Ga-NEB also outperformed conventional ^99m^Tc-SC lymphoscintigraphy in the evaluation of patients with different suspected lymphatic drainage abnormalities [98].

When EB-maleimide, one of the truncated EB derivatives, was conjugated to antidiabetic drug exendin-4 (denoted as Abextide), it showed great potential to treat type 2 diabetic mice due to the significantly extended biological half-life of Abextide through complexation with albumin in situ [25, 26]. This strategy provides possibilities for the development of long-acting therapeutic drugs for other small molecules and biologics [26].

## 5. Toxicity of Evans Blue Dye

EB dye has been widely used for different applications in the clinic and research, but few researchers have considered the probable toxicity of the dye. EB dye can cause delayed death in all mice at doses above 200 mg/kg body weight [99], but the usual dosage of 2% EB, 0.5 mL/kg (10 mg/kg), or even lower causes much less toxicity compared to higher doses. In most cases, few reports of adverse reactions were seen. However, it was reported by Gibson and Gregersen in 1935 that pulmonary embolism was observed after intravenous injection of EB in rats. In 1974, Giger et al. demonstrated that EB can cause platelet aggregation when the molar ratio of EB : albumin is >1 : 1 [100]. Similarly, by the late end of the 20th century, Moos and Mollgard found that free dye (albumin-unbound EB) in plasma caused 60% of neonatal and 20% of the adult animal deaths during their experiments, and the toxicity of free dye led to alteration of the BBB permeability, while it appeared to be safe when EB was used as single intravenous injection at a conventional clinical dose [100, 101]. In addition, Jackiewicz et al. also proved that EB dye caused a significant simplification of the junctional morphology in normal and regenerating endothelium, which should be taken into account when using EB dye to assess various insults to blood vessels, in 1998 [68]. Recently, an ex vivo experiment by Giansanti et al. investigated the safety of EB dye. They found damage of ARPE-19 cells and RGC5 cells with halogen and xenon light exposure should be a concern when the dye is used during vitrectomy [102]. The point that many researchers have easily ignored is that azo dyes, as a class, can potentially induce mutagenicity and carcinogenicity when split off or degraded into component aromatic amines, especially in the fetus. Although there is a lack of reports about the carcinogenicity of EB, it is necessary to consider the possibility when using EB in clinical practice.

## 6. Conclusion

In summary, the once widely used EB dye dilution method has gradually been replaced by newer methods, such as radiolabeled albumin and radiolabeled red cells, for the estimation of blood volume and cardiac output. The blood pool effect of EB dye is limited when evaluating perfused or hypoperfused myocardium, but its utility in assessing vascular permeability and BBB integrity has increased. In addition, the use of radiolabeled EB and its derivatives will play an important role in clinical imaging of tumor lesions, evaluation of lymphatic disorders, and development of long-acting therapeutics. Importantly, the newly discovered necrosis-avid affinity of EB will facilitate the identification of necrotic tissues including myocardial infraction, cerebral stroke, degenerating muscular diseases, and even amyloidosis [103], as well as the assessment of therapeutic efficacy and prognosis of solid tumors. Although the mechanisms are still unclear and further investigation is required, EB dye as a vital stain has greatly contributed to biomedical research and may continue to benefit the development of medical practice and patient care. Therefore, the main advantage of new applications of Evans blue lies in its great potential use in clinical practice as mentioned above.

## Figures and Tables

**Table 1:** Characteristics of different blue dyes.

Dye	Molecular formula	Chemical structure	Number of –SO3H groups	Size (Da)	Max spectral absorption (nm)	% bound to plasma proteins [27]	Time of complete clearance from plasma (minutes) [1]	Applications in biomedicine
Methylene blue	C_16_H_18_ClN_3_S	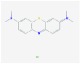	0	320	670	0.0 ± 0.0	<20	Treating methemoglobinemia [4, 27]
Patent blue	C_27_H_31_N_2_O_6_S_2_	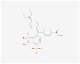	2	567	640	4.7 ± 1.2	<25	Detection of lymph nodes [3]
Trypan blue	C_34_H_24_N_6_Na_4_O_14_S_4_	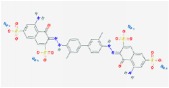	4	961	600	62.2 ± 2.0	<120	Staining biopsies, living cells, and organisms [2]
Evans blue	C_34_H_24_N_6_Na_4_O_14_S_4_	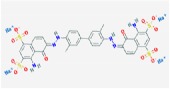	4	961	620	68.1 ± 3.5	>120	Estimation of blood volume, detection of lymph nodes, localization of tumors, and so forth [6, 12, 18]

## References

[1] Davis H. A., al-Fadly W., Gibson L. H. (1958). Use of Dyes with Rapid Bloodstream Clearance for Serial Determination of Cardiac Output. *Proceedings of the Society for Experimental Biology and Medicine*.

[2] Cooksey C. J. (2014). Quirks of dye nomenclature. 3. Trypan blue. *Biotechnic & Histochemistry*.

[3] Cooksey C. J. (2017). Quirks of dye nomenclature. 8. Methylene blue, azure and violet. *Biotechnic & Histochemistry*.

[4] Gelmini R., Campanelli M., Cabry F. (2017). Role of sentinel node in differentiated thyroid cancer: a prospective study comparing patent blue injection technique, lymphoscintigraphy and the combined technique. *Journal of Endocrinological Investigation*.

[5] Evans H. M., Schulemann W. (1914). The action of vital stains belonging to the benzidine group. *Science*.

[6] Cooksey C. J. (2014). Quirks of dye nomenclature. 1. Evans blue. *Biotechnic & Histochemistry*.

[7] Steinwall O., Klatzo I. (1966). Selective vulnerability of the blood-brain barrier in chemically induced lesions. *Journal of Neuropathology & Experimental Neurology*.

[8] Wolman M., Klatzo I., Chui E. (1981). Evaluation of the dye-protein tracers in pathophysiology of the blood-brain barrier. *Acta Neuropathologica*.

[9] Saria A., Lundberg J. M. (1983). Evans blue fluorescence: quantitative and morphological evaluation of vascular permeability in animal tissues. *Journal of Neuroscience Methods*.

[10] Yen L. F., Wei V. C., Kuo E. Y., Lai T. W. (2013). Distinct Patterns of Cerebral Extravasation by Evans Blue and Sodium Fluorescein in Rats. *PLoS ONE*.

[11] Manaenko A., Chen H., Kammer J., Zhang J. H., Tang J. (2011). Comparison Evans Blue injection routes: Intravenous versus intraperitoneal, for measurement of blood-brain barrier in a mice hemorrhage model. *Journal of Neuroscience Methods*.

[12] Roller B. T., Munson J. M., Brahma B., Santangelo P. J., Pai S. B., Bellamkonda R. V. (2015). Evans blue nanocarriers visually demarcate margins of invasive gliomas. *Drug Delivery and Translational Research*.

[13] Zheng M., Yu J., Tian Z. (2013). Characterization of the liver-draining lymph nodes in mice and their role in mounting regional immunity to HBV. *Cellular & Molecular Immunology*.

[14] Smith S. J., Horstick E. J., Davidson A. E., Dowling J. (2015). Analysis of zebrafish larvae skeletal muscle integrity with evans blue dye. *Journal of Visualized Experiments*.

[15] Maeda H., Fang J., Inutsuka T., Kitamoto Y. (2003). Vascular permeability enhancement in solid tumor: Various factors, mechanisms involved and its implications. *International Immunopharmacology*.

[16] Mammoto A., Mammoto T., Kanapathipillai M. (2013). Control of lung vascular permeability and endotoxin-induced pulmonary oedema by changes in extracellular matrix mechanics. *Nature Communications*.

[17] Bábíčková J., Klinkhammer B. M., Buhl E. M. (2017). Regardless of etiology, progressive renal disease causes ultrastructural and functional alterations of peritubular capillaries. *Kidney International*.

[18] Harrell M. I., Iritani B. M., Ruddell A. (2008). Lymph node mapping in the mouse. *Journal of Immunological Methods*.

[19] Caster W. O., Simon A. B., Armstrong W. D. (1954). Analytical Problems in Determination of Evans Blue: Caused by Absorption on Glass and Protein Surfaces. *Analytical Chemistry*.

[20] Lacy W. W., Ugaz C., Newman E. V. (1955). The use of indigo carmine for dye dilution curves. *Circulation Research*.

[21] Lawrence A. C., Walters G. (1959). The extraction of Evans blue (T1824) from plasma and the measurement of plasma volume. *Journal of Clinical Pathology*.

[22] Szabo S., Trier J. S., Brown A., Schnoor J. (1985). Early Vascular Injury and Increased Vascular Permeability in Gastric Mucosal Injury Caused by Ethanol in the Rat. *Gastroenterology*.

[23] Tsopelas C., Bevington E., Kollias J. (2006). 99mTc-Evans blue dye for mapping contiguous lymph node sequences and discriminating the sentinel lymph node in an ovine model. *Annals of Surgical Oncology*.

[24] Radu M., Chernoff J. (2013). An in vivo assay to test blood vessel permeability.. *Journal of visualized experiments : JoVE*.

[25] Liu Y., Wang G., Zhang H. (2016). Stable Evans Blue Derived Exendin-4 Peptide for Type 2 Diabetes Treatment. *Bioconjugate Chemistry*.

[26] Chen H., Wang G., Lang L. (2016). Chemical conjugation of evans blue derivative: A strategy to develop long-acting therapeutics through albumin binding. *Theranostics*.

[27] Tsopelas C., Sutton R. (2002). Why certain dyes are useful for localizing the sentinel lymph node. *Journal of Nuclear Medicine*.

[28] Saunders N. R., Dziegielewska K. M., Møllgård K., Habgood M. D. (2015). Markers for blood-brain barrier integrity: How appropriate is Evans blue in the twenty-first century and what are the alternatives?. *Frontiers in Neuroscience*.

[29] Shen Y. (2014). Fluorescence imaging of Evans blue extravasation into mouse brain induced by low frequency ultrasound with microbubble. *Bio-Medical Materials and Engineering*.

[30] Do J., Foster D., Renier C. (2014). Ex vivo Evans blue assessment of the blood brain barrier in three breast cancer brain metastasis models. *Breast Cancer Research and Treatment*.

[31] Muldoon L. L., Pagel M. A., Netto J. P., Neuwelt E. A. (2016). Intra-arterial administration improves temozolomide delivery and efficacy in a model of intracerebral metastasis, but has unexpected brain toxicity. *Journal of Neuro-Oncology*.

[32] Feng Y., Chen F., Ma Z. (2014). Towards stratifying ischemic components by cardiac MRI and multifunctional stainings in a rabbit model of myocardial infarction. *Theranostics*.

[33] Cona M. M., Koole M., Feng Y. (2014). Biodistribution and radiation dosimetry of radioiodinated hypericin as a cancer therapeutic. *International Journal of Oncology*.

[34] Gibson J. G., Evans W. A. (1937). Clinical studies of the blood volume. I. Clinical application of a method employing the azo dye “evans blue” and the spectrophotometer. *The Journal of Clinical Investigation*.

[35] Dawson A. B., Evans H. M., Whipple G. H. (1920). Blood volume studies. *American Journal of Physiology-Legacy Content*.

[36] Reeve E. B., Allen T. H., Roberts J. E. (1960). Blood Volume Regulation. *Annual Review of Physiology*.

[37] M J. F., Shillingford J. P. (1958). A comparison of direct and extraction methods for the determination of T-1824 (Evans blue) in plasma and serum. *Journal of Clinical Pathology*.

[38] Crooke A. C., Morris C. J. O. (1942). The determination of plasma volume by the Evans blue method. *The Journal of Physiology*.

[39] Hopper J., Tabor H., Winkler A. W. (1944). Simultaneous measurements of the blood volume in man and dog by means of evans blue dye, T1824, and by means of carbon monoxide. I. Normal subjects 1. *The Journal of Clinical Investigation*.

[40] Cruickshank E. W. H., Whitfield I. C. (1945). The behaviour of T. 1824 (Evans's blue) in circulating blood and a modified method for the estimation of plasma volume. *The Journal of Physiology*.

[41] Hobsley M., Dew E. D. (1958). An extraction technique for the estimation of Evans blue in plasma. *Journal of Clinical Pathology*.

[42] Farjanel J., Denis C., Chatard J. C., Geyssant A. (1996). An accurate method of plasma volume measurement by direct analysis of Evans blue spectra in plasma without dye extraction: Origins of albumin-space variations during maximal exercise. *European Journal of Applied Physiology*.

[43] Wang H.-L., Lai T. W. (2014). Optimization of Evans blue quantitation in limited rat tissue samples. *Scientific Reports*.

[44] Margouleff D. (2013). Blood volume determination, a nuclear medicine test in evolution. *Clinical Nuclear Medicine*.

[45] Baby P. M., Kumar P., Kumar R. (2014). A novel method for blood volume estimation using trivalent chromium in rabbit models. *Indian Journal of Plastic Surgery*.

[46] Dingley J., Foëx B. A., Swart M. (1999). Blood volume determination by the carbon monoxide method using a new delivery system: Accuracy in critically ill humans and precision in an animal model. *Critical Care Medicine*.

[47] Yet S., Tian R., Layne M. D. (2001). Cardiac-specific expression of heme oxygenase-1 protects against ischemia and reperfusion injury in transgenic mice. *Circulation Research*.

[48] Lucking E. F., O'Halloran K. D., Jones J. F. X. (2014). Increased cardiac output contributes to the development of chronic intermittent hypoxia-induced hypertension. *Experimental Physiology*.

[49] Stewart G. N. (1897). Researches on the Circulation Time and on the Influences which affect it. *The Journal of Physiology*.

[50] Hamilton W. F., Riley R. L., Attyah A. M. (1948). Comparison of the fick and dye injection methods of measuring the cardiac output in man. *American Journal of Physiology-Legacy Content*.

[51] Witham A. C., Fleming J. W., Bloom W. L. (1951). The effect of the intravenous administration of dextran on cardiac output and other circulatory dynamics. *The Journal of Clinical Investigation*.

[52] García X., Mateu L., Maynar J., Mercadal J., Ochagavía A., Ferrandiz A. (2011). Estimating cardiac output. Utility in the clinical practice. Available invasive and non-invasive monitoring. *Medicina Intensiva (English Edition)*.

[53] Thiele R. H., Bartels K., Gan T. J. (2015). Cardiac output monitoring: A contemporary assessment and review. *Critical Care Medicine*.

[54] Nguyen L. S., Squara P. (2017). Non-Invasive Monitoring of Cardiac Output in Critical Care Medicine. *Frontiers in Medicine*.

[55] Romson J. L., Hook B. G., Kunkel S. L., Abrams G. D., Schork M. A., Lucchesi B. R. (1983). Reduction of the extent of ischemic myocardial injury by neutrophil depletion in the dog. *Circulation*.

[56] Jolly S. R., Kane W. J., Bailie M. B., Abrams G. D., Lucchesi B. R. (1984). Canine myocardial reperfusion injury. Its reduction by the combined administration of superoxide dismutase and catalase. *Circulation Research*.

[57] Bialik S., Geenen D. L., Sasson I. E. (1997). Myocyte apoptosis during acute myocardial infarction in the mouse localizes to hypoxic regions but occurs independently of p53. *The Journal of Clinical Investigation*.

[58] Kurrelmeyer K. M., Michael L. H., Baumgarten G. (2000). Endogenous tumor necrosis factor protects the adult cardiac myocyte against ischemic-induced apoptosis in a murine model of acute myocardial infarction. *Proceedings of the National Acadamy of Sciences of the United States of America*.

[59] Stanton H., Rogerson F. M., East C. J. (2005). ADAMTS5 is the major aggrecanase in mouse cartilage in vivo and in vitro. *Nature*.

[60] Fishbein M. C., Meerbaum S., Rit J. (1981). Early phase acute myocardial infarct size quantification: Validation of the triphenyl tetrazolium chloride tissue enzyme staining technique. *American Heart Journal*.

[61] Miller D. L., Li P., Dou C., Armstrong W. F., Gordon D. (2007). Evans Blue Staining of Cardiomyocytes Induced by Myocardial Contrast Echocardiography in Rats: Evidence for Necrosis Instead of Apoptosis. *Ultrasound in Medicine & Biology*.

[62] Kaya M., Ahishali B. (2011). Assessment of permeability in barrier type of endothelium in brain using tracers: Evans blue, sodium fluorescein, and horseradish peroxidase. *Methods in Molecular Biology*.

[63] Kozler P., Pokorný J. (2003). Altered Blood-Brain Barrier Permeability and Its Effect on the Distribution of Evans Blue and Sodium Fluorescein in the Rat Brain Applied by Intracarotid Injection. *Physiological Research*.

[64] Uyama O., Okamura N., Yanase M., Narita M., Kawabata K., Sugita M. (1988). Quantitative evaluation of vascular permeability in the gerbil brain after transient ischemia using Evans blue fluorescence. *Journal of Cerebral Blood Flow & Metabolism*.

[65] Slama M., Susic D., Varagic J., Ahn J., Frohlich E. D. (2003). Echocardiographic measurement of cardiac output in rats. *American Journal of Physiology-Heart and Circulatory Physiology*.

[66] Roberts W. G., Palade G. E. (1995). Increased microvascular permeability and endothelial fenestration induced by vascular endothelial growth factor. *Journal of Cell Science*.

[67] Huang L., Cao W., Deng Y., Zhu G., Han Y., Zeng H. (2016). Hypertonic saline alleviates experimentally induced cerebral oedema through suppression of vascular endothelial growth factor and its receptor VEGFR2 expression in astrocytes. *BMC Neuroscience*.

[68] Jackiewicz T. A., McGeachie J. K., London R. M., Tennant M. (1998). Evans blue dye modifies the ultrastructure of normal and regenerating arterial endothelium in rats. *Microsurgery*.

[69] Chen F., Suzuki Y., Nagai N. (2004). Visualization of stroke with clinical MR imagers in rats: a feasibility study.. *Radiology*.

[70] Soubeyrand M., Badner A., Vawda R., Chung Y. S., Fehlings M. G. (2014). Very high resolution ultrasound imaging for real-time quantitative visualization of vascular disruption after spinal cord injury. *Journal of Neurotrauma*.

[71] Pettersson C. Å. V., Sharma H. S., Olsson Y. (1990). Vascular permeability of spinal nerve roots - A study in the rat with Evans blue and lanthanum as tracers. *Acta Neuropathologica*.

[72] Xu Q., Qaum T., Adamis A. P. (2001). Sensitive blood-retinal barrier breakdown quantitation using Evans blue. *Investigative Ophthalmology & Visual Science*.

[73] Alves da Silva J. A., Oliveira K. C., Camillo M. A. P. (2011). Gyroxin increases blood-brain barrier permeability to Evans blue dye in mice. *Toxicon*.

[74] Sutton R., Tsopelas C., Kollias J., Chatterton B. E., Coventry B. J. (2002). Sentinel node biopsy and lymphoscintigraphy with a technetium 99m labeled blue dye in a rabbit model. *Surgery*.

[75] Bobin J. Y. (1999). Tagging sentinel lymph nodes: a study of 100 patients with breast cancer. *European Journal of Cancer*.

[76] Tervala T. V., Hartiala P., Tammela T. (2015). Growth factor therapy and lymph node graft for lymphedema. *Journal of Surgical Research*.

[77] Bass S. S. (1999). The role of sentinel lymph node biopsy in breast cancer. *Journal of the American College of Surgeons*.

[78] Ozawa T., Britz G. W., Kinder D. H. (2005). Bromophenol blue staining of tumors in a rat glioma model. *Neurosurgery*.

[79] Maeda H., Nakamura H., Fang J. (2013). The EPR effect for macromolecular drug delivery to solid tumors: Improvement of tumor uptake, lowering of systemic toxicity, and distinct tumor imaging in vivo. *Advanced Drug Delivery Reviews*.

[80] Prabhu S. S., Broaddus W. C., Oveissi C., Berr S. S., Gillies G. T. (2000). Determination of intracranial tumor volumes in a rodent brain using magnetic resonance imaging, Evans Blue, and histology: A comparative study. *IEEE Transactions on Biomedical Engineering*.

[81] Elsen S., Lerut E., Van Cleynenbreugel B., Van Der Aa F., Van Poppel H., De Witte P. A. (2015). Biodistribution of Evans blue in an orthotopic AY-27 rat bladder urothelial cell carcinoma model: Implication for the improved diagnosis of non-muscle-invasive bladder cancer (NMIBC) using dye-guided white-light cystoscopy. *BJU International*.

[82] Roelants M., Huygens A., Crnolatac I. (2012). Evans blue as a selective dye marker for white-light diagnosis of non-muscle-invasive bladder cancer: An in vitro study. *BJU International*.

[83] Zhang X., Xie Y., Jin Y., Hou X., Ye L., Lou J. (2004). The effect of RMP-7 and its derivative on transporting evens blue liposomes into the brain. *Drug Delivery: Journal of Delivery and Targeting of Therapeutic Agents*.

[84] McNeeley K. M., Annapragada A., Bellamkonda R. V. (2007). Decreased circulation time offsets increased efficacy of PEGylated nanocarriers targeting folate receptors of glioma. *Nanotechnology*.

[85] Morera C., Villanueva M. A. (2009). Heat treatment and viability assessment by Evans blue in cultured Symbiodinium kawagutii cells. *World Journal of Microbiology and Biotechnology*.

[86] Jacyn Baker C., Mock N. M. (1994). An improved method for monitoring cell death in cell suspension and leaf disc assays using evans blue. *Plant Cell, Tissue and Organ Culture*.

[87] Matsuda R., Nishikawa A., Tanaka H. (1995). Visualization of dystrophic muscle fibers in Mdx mouse by vital staining with Evans blue: evidence of apoptosis in dystrophin-deficient muscle. *The Journal of Biochemistry*.

[88] Straub V., Rafael J. A., Chamberlain J. S., Campbell K. P. (1997). Animal models for muscular dystrophy show different patterns of sarcolemmal disruption. *The Journal of Cell Biology*.

[89] Klyen B. R., Shavlakadze T., Radley-Crabb H. G., Grounds M. D., Sampson D. D. (2011). Identification of muscle necrosis in the mdx mouse model of Duchenne muscular dystrophy using three-dimensional optical coherence tomography. *Journal of Biomedical Optics*.

[90] Wooddell C. I., Zhang G., Griffin J. B., Hegge J. O., Huss T., Wolff J. A. (2010). Use of Evans blue dye to compare limb muscles in exercised young and old mdx mice. *Muscle & Nerve*.

[91] Hamer P. W., McGeachie J. M., Davies M. J., Grounds M. D. (2002). Evans Blue Dye as an in vivo marker of myofibre damage: optimising parameters for detecting initial myofibre membrane permeability. *Journal of Anatomy*.

[92] Miller D. L., Li P., Dou C., Gordon D., Edwards C. A., Armstrong W. F. (2005). Influence of contrast agent dose and ultrasound exposure on cardiomyocyte injury induced by myocardial contrast echocardiography in rats. *Radiology*.

[93] Yasuda S., Ikuta K., Uwatoku T. (2008). In vivo magnetic resonance imaging of atherosclerotic lesions with a newly developed evans blue-DTPA-gadolinium contrast medium in apolipoprotein-E- deficient mice. *Journal of Vascular Research*.

[94] Ni Y., Bormans G., Chen F., Verbruggen A., Marchal G. (2005). Necrosis avid contrast agents: Functional similarity versus structural diversity. *Investigative Radiology*.

[95] Tsopelas C., Bellon M., Bevington E., Kollias J., Shibli S., Chatterton B. E. (2008). Lymphatic mapping with 99mTc-Evans Blue dye in sheep. *Annals of Nuclear Medicine*.

[96] Niu G., Lang L., Kiesewetter D. O. (2014). In vivo labeling of serum albumin for PET. *Journal of Nuclear Medicine*.

[97] Zhang J., Lang L., Zhu Z., Li F., Niu G., Chen X. (2015). Clinical translation of an albumin-binding PET radiotracer 68Ga-NEB. *Journal of Nuclear Medicine*.

[98] Zhang W., Wu P., Li F., Tong G., Chen X., Zhu Z. (2016). Potential applications of using 68Ga-evans blue PET/CT in the evaluation of lymphatic disorder preliminary observations. *Clinical Nuclear Medicine*.

[99] Taylor S. H., Thorp J. M. (1959). Properties and biological behaviour of coomassie blue. *Heart*.

[100] Giger M., Baumgartner H. R., Zbinden G. (1974). Toxicological effects of Evans blue and Congo red on blood platelets. *Agents and Actions Supplements*.

[101] Moos T., Mollgard K. (1993). Cerebrovascular permeability to azo dyes and plasma proteins in rodents of different ages. *Neuropathology and Applied Neurobiology*.

[102] Giansanti F., Schiavone N., Papucci L. (2014). Safety testing of blue vital dyes using cell culture models. *Journal of Ocular Pharmacology and Therapeutics*.

[103] Abildgaard U. (1966). Rapid Elimination of Evans Blue in Amyloidosis. *Journal of Internal Medicine*.

