# Corrigendum to “Evans Blue Dye: A Revisit of Its Applications in Biomedicine”

**DOI:** 10.1155/2021/9861738

**Published:** 2021-08-28

**Authors:** Linpeng Yao, Xing Xue, Peipei Yu, Yicheng Ni, Feng Chen

**Affiliations:** ^1^Department of Radiology, The First Affiliated Hospital, Zhejiang University School of Medicine, 79 Qingchun Road, Hangzhou, Zhejiang 310003, China; ^2^Department of Radiology, Sanmen County People's Hospital, Sanmen, Zhejiang 317100, China; ^3^Radiology Section, University Hospitals, University of Leuven, 3000 Leuven, Belgium

In the article titled “Evans Blue Dye: A Revisit of Its Applications in Biomedicine” [1], the authors mistakenly cited Yet et al. instead of Jahr J S et al. on page 3. Thus, the sentence “Yet et al. [47] developed a novel approach using HBOC in rabbits in 2001, which is inexpensive and not time-consuming, while Baby et al.” should be corrected as “Jahr J S et al. [47] developed a novel approach using HBOC in rabbits in 2001, which is inexpensive and not time-consuming, while Baby et al.” and the corresponding reference should be corrected as follows [2]:

  [47] Jahr J S, Lurie F, Xi S, et al. “A novel approach to measuring circulating blood volume: the use of a hemoglobin-based oxygen carrier in a rabbit model”, Anesthesia & Analgesia, 2001, 92(3):609-614.

## References

[1] Yao L., Xue X., Yu P., Ni Y., Chen F. (2018). Evans blue dye: a revisit of its applications in biomedicine. *Contrast Media and Molecular Imaging*.

[2] Jahr J. S., Lurie F., Xi S. (2001). A novel approach to measuring circulating blood volume: the use of a hemoglobin-based oxygen carrier in a rabbit model. *Anesthesia & Analgesia*.

